# Eating Habits and Body Weight Changes Induced by Variation in Smell and Taste in Patients with Previous SARS-CoV-2 Infection

**DOI:** 10.3390/nu14235068

**Published:** 2022-11-29

**Authors:** Anna Ferrulli, Pamela Senesi, Ileana Terruzzi, Livio Luzi

**Affiliations:** 1 Department of Endocrinology, Nutrition and Metabolic Diseases, IRCCS MultiMedica, 20138 Milan, Italy; 2 Department of Biomedical Sciences for Health, University of Milan, 20133 Milan, Italy

**Keywords:** COVID-19, smell, taste, eating behaviour, body weight

## Abstract

Olfactory and gustatory dysfunction are recognized as common symptoms in patients with COVID-19, with a prevalence ranging, respectively, between 41–61% and 38.2–49%. This review focused on relating the variations in dietary habits with the reduction/loss of smell and/or taste in patients who contracted the COVID-19 infection. Primarily, we reviewed the main pathological mechanisms involved in COVID 19-induced anosmia/dysosmia and ageusia/dysgeusia. Then, we explored and summarized the behavioural changes in food intake and body weight during the COVID-19 pandemic in relation to sensory impairment and the underlying mechanisms. Most studies on this topic argue that the altered chemosensory perception (taste and smell) mainly induces reduced appetite, leading to a faster fullness sensation during the consumption of a meal and, therefore, to a decrease in body weight. On the other hand, a reduced perception of the food’s sensory properties may trigger compensatory responses that lead some individuals to increase food intake with a different effect on body weight. Regarding body weight, most studies evaluated malnutrition in patients hospitalized for COVID-19; more studies are warranted to investigate nutritional status specifically in non-hospitalized patients with olfactory and gustatory dysfunctions caused by COVID-19 infection.

## 1. Introduction

Coronavirus disease 2019 (COVID-19), caused by the SARS-CoV-2 virus, was responsible for a pandemic in March 2020, leading to a global health crisis [1] that has not yet ended. Among the wide-ranging clinical manifestations of COVID-19, the loss of smell and taste has been widely reported [2]. While at the beginning of the pandemic only few studies described definite changes in smell and taste perception in patients with COVID-19, currently anosmia and dysgeusia are recognized as “significant symptoms” that can occur even in the absence of the “usual symptoms” such as fever, cough, and respiratory failure [3]. In fact, a recent European multicentre study reported smell and/or taste dysfunctions in more than 80% of COVID-19 patients [4]. Several mechanisms seem to be implicated in the pathogenesis of these sensory dysfunctions, mainly linked to the neurotropic and neuro-invasive effects of SARS-CoV-2, by affecting both peripheral neurons and the central nervous system (CNS) [3].

At the same time, variations in feeding behaviour and in body weight control frequently occurred during the nationwide COVID-19 lockdown; initially, they were ascribed mainly to lifestyle changes (e.g., telework and/or online learning, staying at home as much as possible, essential work, limited physical activity and purchasing food) [3,5], but a possible impact of hyposmia/dysosmia and of hypogeusia/dysgeusia on food habits and energy intake cannot be excluded [3], as has been observed in other pathological conditions (e.g., chemotherapy-treated cancer, Parkinson disease…) [6,7].

The aims of the present review are to explore and summarize the behavioural changes in food intake during the COVID-19 pandemic in relation to smell and taste impairment and how the underlying mechanisms through which COVID-19-induced olfactory and gustatory changes could lead to variations in eating habits and body weight.

## 2. Definitions of Smell, Taste, and Flavour

Before delving into the main purpose of the review, it is useful to briefly clarify the terminology relating to the sensory system that is used throughout the manuscript. The sense of smell, or olfaction, or olfactory sensation, is defined as “the perception of an odour or scent of through stimuli affecting the olfactory nerves” [8,9]. It occurs when an odour binds to a receptor within the nasal cavity, transmitting a signal through the olfactory system. The smell is implicated in detecting desirable foods, hazards, and pheromones, and it affects the taste. The sense of taste or gustatory sensation means “the ability to perceive and distinguish the sweet, sour, bitter, salty, or umami quality of a dissolved substance which is mediated by taste buds on the tongue” [10,11]. Taste, along with olfaction and trigeminal nerve stimulation (registering texture, pain, and temperature), determines flavours of food and other substances. Sometimes, taste and flavour are wrongly used as synonyms. According to the definition provided by Spence et al. [12], flavour is defined as a “complex combination of the olfactory, gustatory, and trigeminal sensations perceived during tasting. The flavour may be influenced by tactile, thermal, painful and/or kinaesthetic effects” [from International Standards Organization (ISO 5492,1992, 2008)]. Therefore, Spence et al. sustained that taste should be classified as a sub-component of flavours [12].

## 3. Hints about the Influence of Smell and Taste on Food Control

Food choice and food intake are guided by the sense of both taste and smell, as well as by metabolic processes.

The mechanisms underlying olfaction involve a self-regenerating olfactory epithelium, composed by millions of olfactory sensory neurons (OSNs). Axons of OSNs reach the glomeruli in the olfactory bulbs to form synapses through the cribriform plate in the superior part of the nasal cavity.

Olfactory sensations are mediated by numerous multigene olfactory receptors (ORs), which are members of the guanine nucleotide protein (G protein)-coupled receptors (GPCRs) superfamily and their activation induces ATP conversion into cAMP by adenylate cyclase [13]. cAMP triggers the olfactory-specific cyclic nucleotide-gated channel (CNG) [14] and subsequently modifies calcium and chloride efflux, promoting OSNs depolarization [15]. In this manner, odorant information is transmitted not only to the olfactory bulb but also to other brain regions, including the piriform cortex, amygdala, olfactory tubercle, up to OFC, hypothalamus, thalamus, and hippocampus [3,16].

These neuronal connections could explain why sensory exposure to food and food-related cues, beyond its simple hedonic value, affects the food choice, the optimum development of satiation, and the energy intake regulation [16]. Specifically, the smell seems to be involved in the short-term regulation of food intake since it influences mainly the detection of foods and the direction of choices [17], according to the awareness or intensity of the odours or personality traits of the individuals [18].

In recent years, taste basic research has rapidly improved, and taste signaling transduction pathways are partially clarified. Thousands of taste buds are located on the tongue, soft palate, and epiglottis. The taste receptor cells (TRCs), called Type I, II, III, and IV, have been identified and characterized [19]. Type I cells, known as glia cells, collaborate with taste, signaling transduction and primarily removing extracellular neurotransmitters [20]. Type IV cells, also known as precursor cells, differentiate into the other cell types during fast cell turnover in taste buds [21]. Type II cells, receptor cells, are characterized by the expression of GRCRs and are encoded by taste 1 receptor members 2 and 3 (TAS1R2 + TAS1R3) genes [22]. These receptors transduce three basic tastes, sweet, umami, and bitter, by phospholipase C-cascade activation that induces Ca^2+^ release from the stores and culminates into ATP release [23]. It is important to note that recent in vitro and in vivo studies have suggested that different signaling independent GPCPs are involved in sweet transduction in taste cells. This alternative pathway is related to glucose influx; the activation of glucose transports (GLUTs) and the sodium–glucose cotransporters (SGLTs) family increases ATP and subsequently inhibits ATP-sensitive potassium channel (K_ATP_)-dependent signaling [24]. In any case, Type II cells transmit tastes to the afferent neurons via a channel synapse. Type III cells are responsible for the ionic tastes of salty and sour, through the proton selective channel, otopterin1, and conventional synapse mechanisms, such as neurons. For these reasons, Type III cells are also called presynaptic cells [25].

From the taste TRCs, taste information converges to the nucleus of the solitary tract (NTS) through the branch of the facial nerve (chorda tympani), the glossopharyngeal nerve, and the vagus nerve [3]. As well as for smell, taste information reaches several brain regions, including the thalamus and the primary gustatory cortex, located within the somatosensory cortex [3]. Taste provides vital information about (macro) nutrient quality and acceptability [17]. In turn, taste quality is closely associated with palatability. For example, adding specific tastes to foods, such as sweetness and saltiness, increases palatability [17]. Taste is involved in determining the oral exposure duration of food in the mouth and the eating rate, thereby contributing to satiation.

## 4. Potential Mechanisms Involved in COVID 19-Induced Anosmia/Dysosmia

Several mechanisms, alone or in concert, are hypothesized to be involved in COVID-19-induced anosmia/dysosmia [26]. First, nasal congestion or edema of the nasal respiratory epithelium due to viral infection could induce conductive and obstructive damage, leading to anosmia, such as has been observed in the common cold, caused by the endemic strains of human Coronavirus (HCoV) [26]. The hypothesis of the conductive olfactory dysfunction is sustained by the consequent or concomitant onset of anosmia with other ear nose throat (ENT) manifestations and by its recovery within 8 days in the majority of COVID-19 patients [4].

Another hypothesis is linked to the olfactory epithelium disruption following COVID-19 infection. In this case, the anosmia/dysosmia could persist for weeks or months after the remission of other ENT symptoms. Several studies showed ACE2 expression in the olfactory epithelium, specifically in the non-neuronal cells (supporting cells, stem cells, and perivascular cells) [27]. Sustentacular cells, a group of cells that play a supportive role for olfactory sensory neurons (e.g., structural and metabolic support), represent the main entry point of SARS-CoV-2 in the olfactory epithelium [28], as they are equipped with the largest expression of ACE2 and the transmembrane serine protease 2 (TMPRSS2) [29], unlike the OSNs. In fact, TMPRSS2 is a cell surface protein primarily expressed by type II pneumocytes in the lung, but also by nasal goblet secretory cells, the small intestine, and prostate glands.

The TMPRSS2 gene, as well as ACE2, encodes for a protease that promotes viral infections and, therefore, is implicated in the pathogenesis of SARS-CoV-2.

Since the COVID-19-induced-damage of the nasal mucosa is usually repairable, the long-term persistence of anosmia, experienced by numerous individuals, may not be explained by this.

Based on the persistence of anosmia/dysosmia in subjects infected with SARS-CoV-2, CNS involvement through the retrograde propagation of the virus to higher-order neurons is hypothesized [26]. In the past, a model of retrograde olfactory neuroinvasion as the cause of anosmia was described for the Herpes virus [30]. The Herpes virus seems to spread through the SNC via the olfactory and trigeminal nerves, in some cases causing encephalitis, which in turn can lead to permanent anosmia and other neurological complications (epilepsy, amnesia, and cognitive deficits). Armier et al. [31] investigated both acute and chronic manifestations of Herpes Simplex Encephalitis (HSE) in animal models, showing, in the first case, necrotic debris with inflammatory infiltrate (neutrophils, macrophages, and lymphocytes) in the olfactory bulb and, in the last case, severe atrophy of the piriform and entorhinal cortices and amygdala [31]. Based on such evidence, severe anosmia/dysosmia in patients with COVID-19 has been hypothesized to be induced by a central pattern of olfactory impairment involving limbic areas. Recently, a neuroimaging study of SARS-CoV-2, involving 785 participants who were imaged twice using magnetic resonance imaging (MRI) (before and after SARS-CoV-2 infection), revealed a significant impairment mainly of the limbic and olfactory cortices. For example, tissue damage in regions that are functionally connected to the piriform cortex, olfactory tubercle, and anterior olfactory nucleus, as well as a greater reduction in grey matter thickness in the OFC and para-hippocampal gyrus, occurred [32].

Radiological abnormalities found in the olfactory system, specifically in the olfactory bulb, of patients with COVID-19 have been confirmed also at an ultrastructural level. In a post-mortem study, activated microglia adjacent to neurons was found in five patients dead from COVID-19, suggesting the onset of neuronophagia in the olfactory bulb, substantia nigra, and dorsal motor nucleus of the vagal nerve [33]. Recently, a multicentre post-mortem study involving 23 deceased patients with COVID-19 and 14 control individuals showed that COVID-19 infection is associated with axon injuries and microvasculopathy in the olfactory tissue, likely induced by focal or perivascular infiltrates. Together, these findings indicate that olfactory dysfunction in COVID-19 infection may be severe and permanent [34].

Another plausible mechanism of SARS-CoV-2 entry into the CNS is its hematologic spread to endothelial cells of the blood-brain barrier, causing pericyte and astrocyte damages. This hypothesis is supported by the finding of ACE2 expression in the perivascular cells of the olfactory epithelium [27].

## 5. Potential Mechanisms Involved in COVID 19-Induced Ageusia/Dysgeusia

Despite numerous hypotheses about COVID-19-related taste loss, far fewer studies have objectively documented the loss of taste than that of smell. One of the first studies, in which COVID-19-related chemosensory dysfunctions were detected and quantified by specific tests in healthcare workers (for taste, the Brief Self-administered Empirical Taste Test), showed lower olfactory scores in individuals with recent SARS-CoV-2 infection but equivalent gustatory scores compared to other subjects [35]. In another study in COVID-19 patients, the subjective loss of smell was confirmed by a hyposmic test result in 72%, whereas the subjective loss in taste was confirmed by a hypogeusic test in only 33%, suggesting that COVID-19 is tightly associated with olfactory loss but not with gustatory dysfunction when tested physically and not only psychologically [36]. This discrepancy could be explained by the frequent identification of loss of smell with loss of taste, mostly caused by impaired retronasal olfaction [12,37], which is perceived as an alteration of taste.

COVID-induced ageusia/dysgeusia has not been extensively investigated by the scientific community, probably because this symptom has mostly been overlooked compared to other more serious ones. Multiple hypotheses have been proposed to explain the mechanisms underlying the effects of SARS-CoV-2 on gustatory sense.

ACE2, expressed on the epithelial cells and on taste bud cells, obviously represents the main entry point of SARS-CoV-2, together with the sialic acid receptors and the toll-like receptors (TLR) [38,39]. A front-line mechanism contributing to taste loss could be the interference between the binding of SARS-CoV-2 to salivary sialic acid and the glycoproteins-mediated transport of tastants [40].

Ageusia/dysgeusia could also be the result of the impaired renewal of taste buds (which usually have a fast turnover), following the cytokine storm induced by SARS-CoV-2; this effect could be mediated by TLR and interferon (IFN) receptors, which are highly expressed in taste buds, and their activation may limit taste cell regeneration [41], resulting in the extinction of their function.

Furthermore, taste bud cells express multiple entry receptors, which make them highly susceptible to SARS-CoV-2 infection [39]. The direct infection of taste bud cells by the virus and the inflammatory response affect taste perception [39]. A key role of interleukin-6 (IL-6) in the pathogenesis of taste disorder in COVID-19 patients has been highlighted [42]. The active process of taste bud renewal seems to be inhibited by the high levels of proinflammatory cytokines IL-6 and TNF-α, reducing the lifespan of mature taste bud cells via the activation of the apoptotic pathway [43,44]. In turn, inflammation may increase epithelial cell exfoliation, leading to greater sources of viral RNA in saliva [39].

Such as for the anosmia, a SARS-CoV-2-induced impairment of the peripheral and CNS may be hypothesized as possible mechanism for dysgeusia. Among the cranial nerves (CNs) responsible for gustation (CN VII, IX, or X), damage to the chorda tympani (CN VII) might be the most plausible explanation. After the colonization of the nasopharynx, SARS-CoV-2 reaches the middle ear through the eustachian tube, leading to the subsequent damage of the chorda tympani and then to dysgeusia [45].

At the CNS level, a possible effect on taste could be mediated by IL-6; this cytokine, by targeting the thermo-regulatory centre in the hypothalamus during COVID-19 infections, can affect the nearby thalamus, where both the gustatory and the olfactory pathways converge [42,44]. Based on the previously mentioned study by Douaud et al. [32], the hypothesis of the functional and structural impairment of the brain areas that regulate the sense of taste cannot be excluded. The authors found more pronounced SARS-CoV-2-induced tissue damage in the OFC and anterior cingulate cortex, as well as in the insula and the amygdala. Insula, especially the anterior insula, contains the gustatory cortex, and it is connected to the primary olfactory cortex. In turn, the dorsal part of the insula is connected to the OFC, which plays a crucial role in encoding affective value and the computation of perceived pleasantness of taste [46], and in integrating sensory and limbic cues to target feeding behaviour [47].

A scientific debate is ongoing as to whether anosmia/dysosmia and ageusia/dysgeusia often precede full-blown COVID-19 disease or if they are sometimes the only symptoms; thus, they are unlikely to be the result of CNS impairment. On the other hand, it has also been speculated that these symptoms, if caused by the direct loss of olfactory neurons or the damage of the olfactory epithelium or taste bud cells, may lead to a loss of grey matter in the olfactory/gustatory-related brain regions through repeated sensory deprivation [32].

## 6. Variations in Eating Habits and Body Weight Due to COVID-19-Induced Smell and Taste Changes

In the last two years, a great deal of studies investigated changes in dietary habits and body weight in the general population during the COVID-19 pandemic. Most of them agreed that people have increased their food consumption on average, their consumption of high-calorie foods, and their body weight. A recent survey reported that 60% of adults gained a mean of 5.6 kg in body weight [48]; a smaller weight gain (about 1.57 kg) has been suggested by a meta-analysis [49]. A more recent study showed a significant increase in body weight (about 3 kg), body mass index (BMI), diastolic blood pressure, total cholesterol, and a change in eating habits (enhanced fat/oil intake and reduced fruit intake) compared to a pre-COVID control group [50]. Conversely, a cohort study involving 102,889 adults of several races and ethnicities (mean age 56.4 years, mean BMI 30.8 kg/m^2^) treated in outpatient settings found that changes in body weight and BMI, seen after the COVID-19 lockdown, were not significantly greater than those occurring during the pre-lockdown period [51]. These differences between studies may be explained by different methodologies in data collection; in fact, the last study was not based on self-reported data and self-selected patients, but it was based on data collected in outpatient settings and was compared with data obtained in the pre-COVID period [51].

Notwithstanding, the studies on eating habits and body weight changes in individuals who became ill with SARS-Co-V-2 are less numerous than the ones carried out in the general population. The impact of olfactory and gustatory dysfunctions on diet and, to a lesser extent, on body weight has been documented enough, in view of the considerable prevalence of these symptoms.

### 6.1. Changes in Eating Habits in Individuals with Smell and Taste Dysfunctions Due to COVID-19

One of the first studies investigating this topic was based on social media posts of individuals with post-COVID-19 alterations in taste and smell, from March 2020 to September 2020 [52]. The Facebook group “AbScent COVID-19 Smell and Taste Loss”, which included over 9000 active members, was created. Group members were asked to detail their personal journey with smell loss, to describe changes in their experience of odour, and to describe how the change in smell affected their relationship with food and their body weight. In cases where smell and taste did not recover within 2–3 weeks, and it lasted for months, participants reported: a reduced desire and ability to eat and prepare food, weight gain, weight loss and nutritional insufficiency, even impaired psychological status (reduced emotional wellbeing, altered intimacy, and altered relationship to self and others) [52].

In a more recent study, twenty subjects (eighteen women and two men) who experienced chemosensory loss associated with SARS-CoV-2 infection underwent a semi-structured interview, which consisted of several open questions focused on five major themes concerning the nature of altered chemosensory perception and consequent changes in appetite, experiences of eating, eating behaviour, and well-being [53]. The study results were not quantitative but qualitative. All participants experienced altered smells, reporting mostly anosmia or hyposmia. Only one participant experienced parosmia, the altered perception of familiar odour, and then phantosmia, the perception of smell in the absence of an odour. Loss of taste was less common (only five participants). Anosmia caused a decreased appetite and reduced enjoyment related to food and drinks. Most participants reported adopting compensatory strategies for the diminished food flavour to maintain the “food liking”: choosing salty, sweet, and spicy foods and increasing their attention to food texture. The “wanting” component of food reward, related to the post-ingestion detection of nutrients during the meal, was unchanged (the participants referred to this as “mindless eating”) and accounted for a bidirectional trend in body weight change by inducing both the loss and increase of weight (only six participants reported weight loss). An increase in alcohol intake was mentioned by some participants, in part due to the reduced intensity of the perception of alcohol’s disliked flavour [53]. The generalisability of the study’s findings was limited due to the low number of participants involved.

A Danish study investigated the effects of COVID-19 on self-reported appetite (desire for food, hunger, satiety sensation), sensory perception (smell, taste, and flavour), and eating behaviour (meals and intake of food types) [54]. A total of 112 adult participants, who experienced sensory alteration due to COVID-19 disease, underwent an online survey at different stages of recovery from COVID-19: (1) the acute phase (currently ill); (2) the post-acute phase with long-term effects; (3) the post-acute phase (full recovery from COVID-19 disease). The response variables were evaluated by using a three- or five-point categorical scale, with the ends indicating opposite extremes, according to the type of question. Concerning the effects of COVID-19 on appetite, the results of this study confirmed the following previous evidence: lack of hunger sensations was reported as the main cause of reduced appetite in both acute (86% of participants) and post-acute phases (57% of participants). Changes in chemosensory perception (taste and smell) was described as the main cause of alteration in appetite, leading to a faster fullness sensation during the consumption of a meal; in fact, the “liking” of sensory properties triggers a positive feedback mechanism during the early stage of meal and drives continued food intake [55].

Conversely, a reduced perception of the food’s sensory properties may cause less satisfaction after a meal, triggering compensatory responses that lead some individuals to increase their food intake to satisfy these desires (“hedonic” properties of food). In another qualitative study, although the majority of recovering COVID-19 patients experienced decreased appetite and, consequently, decreased food intake, a smaller group of participants experienced an unsatisfied appetite, resulting in a constant search for food and, thus, increased food intake [56]. In this study, data were obtained through a semi-structured interview, based on questions exploring changes in appetite, sensory perception ability, and food-related pleasure.

About the effects of COVID-19 on smell perception in the study by Chaaban et al. [54], participants experienced, in the order of greater frequency: anosmia, parosmia, hyposmia, and, lastly, hyperosmia. Concerning taste, the order of more frequent experiencing was ageusia, hypogeusia, and, lastly, hypergeusia. Alterations were found for all taste attributes (sweet, salty, sour, and bitter); however, during the post-acute phase of COVID-19, an altered ability to perceive sweet and bitter tastes, compared to salty and sour tastes, seemed to last [54,57]. This finding was confirmed by a more recent Italian study [58] that evaluated smell and taste functions in 61 hospitalized COVID-19 patients compared to a control group. Olfactory function was measured by a 12-item odour identification task. The participants were asked to identify the odour; for each odour, liking, intensity, and irritation were measured on a 9-point scale. A taste strip method was used to evaluate gustatory function; the participants were asked to identify the taste after sucking the paper strip and to score intensity on a 9-point scale. About 45% of patients self-reported olfactory and/or gustatory dysfunctions; specifically, sweet and bitter tastes were shown to be more impaired in comparison to other basic tastes. Sweet and bitter tastes share several common mechanisms in receptor signal transduction (i.e., GPCRs); therefore, the authors hypothesized that, at the level of the taste bud cells, the expression and function of the sweet and bitter receptors could be modified by SARS-CoV-2 infection, and, especially, GPCR may also be a possible target of the virus [57].

The study by Chaaban et al. was one of the few that also investigated the effects of COVID-19 on eating behaviour. As a result of decreased appetite, participants declared reduced food intake at every daily meal, reporting a smaller portion size or not eating at all. A preference was expressed for the three main meals (breakfast, lunch, and dinner), compared to snack meals. During the acute phase of COVID-19, differences in the choice/exclusion of some food categories were also observed compared to the pre-COVID-19 period. Specifically, the intake of meat, seafood, eggs, coffee/tea, and salty and sweet snacks was reduced due to a greater awareness of the texture/consistency of this food category, which was not perceived as pleasant [54]. Among the sensory properties, flavour is considered the main driver for the choice of food, especially for a hedonic eating experience; when taste and smell are impaired, other sensory properties of food, especially texture, become drivers of food acceptance. The authors concluded that the consumption of foods with different textures (crunchy foods such as raw ones) or chemesthesis (spicy foods) could be useful to counteract olfactory and gustatory dysfunctions and to improve appetite and eating pleasure [54].

COVID-19-associated olfactory dysfunction is frequently linked with the development of parosmia [52,59].

Parosmia is defined as a “qualitative disorder which alters the individual’s perception of odours in such a way that smells are commonly described as distorted” [60]. Smell distortions are often associated with strong dislike or disgust and sometimes persist for up to 10 years [61]. In a recent cross-sectional study involving 727 individuals, subjects who experienced smell distortions during SARS-CoV-2 infection completed a questionnaire covering aspects of smell loss, parosmia, and the associated changes in valence of everyday items. A significant correlation between strength and disgust (*p* < 0.0001) was found, and when the selected items were reported as distorted, they were mostly (84% of cases) described as unpleasant or gag-inducing [60]. Analogies were made to the rotten, earthy, burnt, or chemical smell; many participants reported a “novel odour”. However, the study showed that not all distorted smells were found to be unpleasant (“euosmia”); sometimes, patients reported the odour of a rose (50% of the time), apple (31%), and butter (29%). Parosmia can lead to changes in eating behaviours by modifying the valence associated with the loss of pleasure expected from food intake. Moreover, parosmia is more frequently associated with an impaired quality of life compared to patients with quantitative olfactory dysfunction [62,63].

### 6.2. Changes in Body Weight in Individuals with Smell and Taste Dysfunctions Due to COVID-19

Whilst a large literature has documented malnutrition in patients hospitalized for COVID-19 mainly due to prolonged hospitalization and immobilization, reduced mobility, catabolic changes particularly in the skeletal muscles, reduced food intake, older age, and hyper-inflammation status [64,65], few studies have investigated nutritional status in patients with COVID-19 managed at home.

In the previously cited study by Burges Watson et al. [52], exploratory research that constituted a “snapshot” of the impact of smell and taste loss on physical health and weight loss was commonly reported, but it was not the exclusive effect. For some participants, flavour loss shifted preferences towards increasing food intake as it “takes more to hit the spot” and, therefore, in weight gain [52]. In another qualitative study, including 20 participants who experienced chemosensory loss associated with COVID-19, only six participants reported weight loss [53]. However, they were among the first studies on this topic, based on self-reported experiences by the participants, and no statistical analysis of data was performed.

Among the more structured studies, a prospective observational study involving 407 hospital-admitted COVID-19 patients (60% at the Intensive Care Unit (ICU) and 40% at the nursing ward), highlighted a serious acute weight loss (>5 kg) in 22% of the patients during their hospital stay at any point in time, especially in the patients admitted to the ICU (85%), and a high risk of sarcopenia (about 73%) in patients during hospital admission [66]. Although the causes that could compromise the nutritional status during SARS-CoV-2 infection are numerous, the high rate of sensory impairments, reported by nursing ward patients, suggested that these symptoms could have serious repercussions on nutritional status. In fact, the most reported complaint was a decreased appetite (58%). One in three patients experienced changed taste, loss of taste (33%), and/or loss of smell (27%). Symptoms of sensory impairment were self-reported.

A post-hoc analysis study evaluated the incidence of unintentional body weight change and malnutrition in 213 COVID-19 patients who were either hospitalized or managed at home and were re-evaluated after clinical remission [67]. The authors found that about 30% of participants lost more than 5% of their baseline body weight; higher systemic inflammation, impaired renal function, and longer disease duration were reported in patients who lost body weight compared to the other participants who did not lose it. For multivariate analyses, only disease duration and, in hospitalized patients, length of stay were significant predictors of weight loss [67], suggesting exposure to inflammation and disease severity as major determinants of body weight loss. The role of alterations in smell and taste in inducing malnutrition was hypothesized, although there was no significant difference in the incidence of hyposmia and hypogeusia between patients who lost weight and those who did not (respectively, for hyposmia: 40.4% vs. 37.1%, *p* = 0.68; for hypogeusia, 46.2% vs. 41.9%, *p* = 0.60) [67].

Although the weight change response to SARS-CoV-2 infection is not one-directional, especially for non-hospitalized infected subjects, weight loss rather than weight gain seems to prevail as a consequence of anosmia/ageusia-induced lack of appetite. However, some evidence of weight gain has been reported. Kaggwa et al. [68] reported a case of severe polyphagia that led to excessive weight gain (BMI rose to from 22 kg/m^2^ to 30 kg/m^2^) in a 41-year-old female with post-acute COVID-19 syndrome. After a mild episode of acute COVID-19 pneumonia, the patient with a negative past medical history experienced increased appetite and the inability to be satisfied and failed to control their food craving. The authors hypothesized CNS involvement and impairment due to a retrograde propagation of SARS-CoV-2 to higher-order neurons [69]. The degeneration of neuronal and glial cells can lead to the damage of important pathways that control appetite.

## 7. Discussion

In this review, after describing the potential mechanisms involved in COVID-19-induced anosmia/dysosmia and/or ageusia/dysgeusia, we explored and summarized the behavioural changes in food intake and body weight variations during the COVID-19 pandemic in relation to sensory impairment.

First, it emerged that chemosensory dysfunctions constitute one of the chief symptoms of SARS-CoV2 infection and can have a significant impact on eating habits and the nutritional status of affected individuals. Nevertheless, since smell and taste impairments are not life-threating conditions, often they are considered secondary or less important problems. The pathological mechanisms underlying smell and taste impairments concern various levels and, according to the level, present a different degree of severity. They may involve the nasal mucosa with the olfactory epithelium or the taste buds, peripheral nerves such as the olfactory and glossopharyngeal nerves, and finally, the CNS. Neuroimaging studies disclosed SARS-CoV-2-induced tissue damage in the OFC and anterior cingulate cortex, as well as in the insula and the amygdala; these brain areas play a crucial role in integrating sensory and limbic cues to target feeding behaviour.

In most cases, changes in chemosensory perception (taste, smell, and flavour) represent the main cause of alteration in appetite, leading to a faster fullness sensation during the consumption of a meal and, therefore, to a reduced food intake. On the other hand, a reduced perception of the food’s sensory properties may cause less satisfaction after a meal, triggering compensatory responses that lead some individuals to increase food intake to satisfy these desires (“hedonic” properties of food). This different attitude towards food can be translated, respectively, into a reduction (more frequently reported in the literature) or an increase in body weight in patients with post-COVID-19 syndrome.

Concerning taste, most studies agree that the most common gustatory alterations concern the perception of sweet and bitter tastes. These sensory alterations affect food choices; in fact, to cope with the changes in appetite, subjects who experienced smell and taste perception tend to satisfy more well-functioning senses, such as touch. This explains the increased intake of spicy, healthy, and crunchy foods. However, despite numerous hypotheses about COVID-19-related taste loss, fewer studies have objectively documented the loss of taste than that of smell. This difference could be explained by the frequent identification of smell loss with taste loss, mostly caused by the retronasal passage of odours, which are perceived as an alteration of taste. Further research is needed to ascertain taste impairment due to COVID-19 infection through objective testing.

About the impact of COVID-19-induced sensory impairment on body weight changes, most studies evaluated malnutrition in patients hospitalized for COVID-19; more studies are warranted to investigate nutritional status specifically in connection with olfactory and gustatory dysfunction induced by COVID-19 infection.

Finally, studying the loss of smell and taste due to COVID-19 and its relationship with impairments in the peripheral and central nervous system offers the opportunity to identify other possible mechanisms involved in dysregulated eating behaviour in obesity. Already, some studies, outside the context of the pandemic, have suggested that defective smell and taste may predispose to obesity, providing a tantalizing clue regarding the potential association of altered smell with obesity in general population [70], by representing a new potential target for possible anti-obesity treatments.

## Data Availability

Not applicable.
